# Biomimetic Constructs for Achilles Tendon Regeneration and their Translation to Human Medicine

**DOI:** 10.1007/s13770-026-00799-0

**Published:** 2026-03-15

**Authors:** Emine Berfu Ozmen, David E. Anderson, Andrew Ward, Madhu Dhar

**Affiliations:** 1 Regenerative Medicine and Tissue Engineering, Department of Large Animal Clinical Sciences, College of Veterinary Medicine, University of Tennessee, Knoxville, TN 37996 USA; 2 Genome Science and Technology, The Bredesen Center for Interdisciplinary Research and Graduate Education, University of Tennessee Knoxville, Knoxville, TN 37996 USA; 3 College of Nursing, University of Tennessee Knoxville, Knoxville, TN 37996 USA; 4 Division of Surgical Oncology, Department of Surgery, University of Tennessee Medical Center, Knoxville, TN 37920 USA

**Keywords:** Biomimetic constructs, Achilles tissue engineering, Clinical translation

## Abstract

**Background:**

Achilles tendon injuries are among the most common lower-body tendon injuries, often resulting fromoveruse and repetitive motion. Current treatments, ranging from conservative therapies to biological grafts, have drawbacks, including limited regenerative capacity and the risk of graft rejection. To overcome these challenges, tissue engineering shows growing promise for Achilles tendon regeneration, with ongoing research focused on developing biomimetic constructs that utilize various materials and biologics. Since every tendon has unique biomechanical and physiological characteristics, it is crucial to conduct individualized evaluations through detailed research and to address key considerations for clinical translation.

**Methods:**

This review focuses explicitly on advances in Achilles tendon tissue engineering for human medicine, assessing constructs made from natural, synthetic, and composite materials with/without biologics and discussing their clinical translation. Studies were searched in the PubMed database and Google Scholar, using the most relevant keywords, such as “Achilles tissue engineering”, “Achilles biomimetic constructs”, “Biomaterials for Achilles tendon”, and “Clinical translation”.

**Results:**

Biomimetic constructs developed from various polymers and their combinations, when integrated with stem cells, demonstrate promising potential to reconstruct tissue microenvironments in vitro and to facilitate tissue repair and biomechanical functions in vivo. Carefully developing each element, including appropriate material structures, is essential for optimizing cell responses, biomechanical properties, and tissue repair in the Achilles tendon. Although the in vitro and in vivo advances reviewed in the paper contribute to clinical research, further studies with reproducible, long-term outcomes are needed to make the constructs clinically applicable in human medicine.

**Conclusion:**

Achilles tissue engineering continues to progress, driven by a deeper understanding of the injuries and the integration of regenerative tools. Furthermore, clinical considerations, such as long-term in vivo follow-up to assess biocompatibility and functional recovery, will be critical to achieving clinical outcomes.

**Graphical Abstract:**

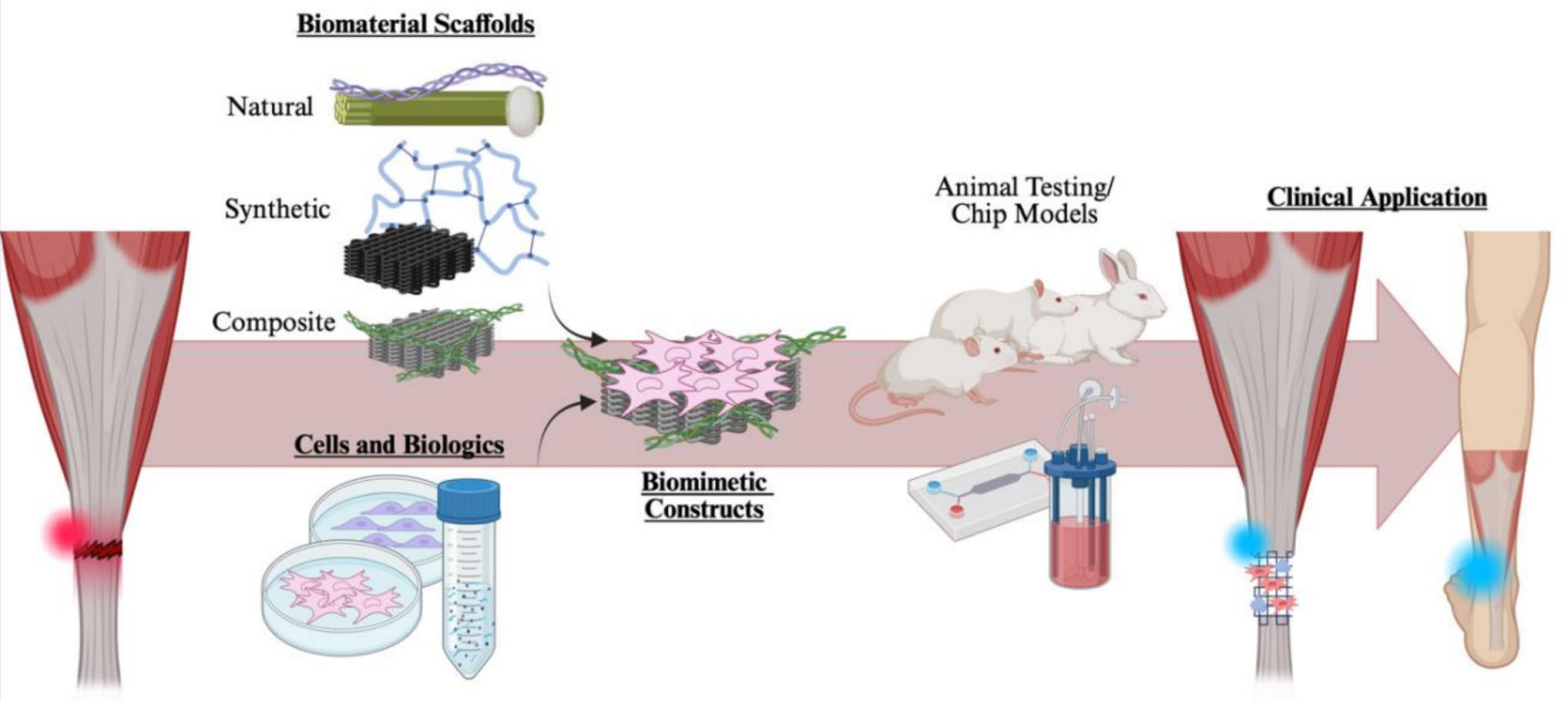

## Introduction

Tendon injuries, including tears and ruptures, are a common form of tendinopathy and are among the most prevalent orthopedic injuries [1]. Annually, millions of people worldwide suffer from tendon injuries, which are common among both athletic and non-athletic populations [2]. The etiology of tendon injuries involves factors such as sports, trauma, overuse, inflammation, age, and genetics [3]. These injuries present significant clinical challenges in patients due to the tendon’s inherent limited regenerative capacities, which are generally associated with the tendon’s hypocellular and hypovascular nature [4], native healing processes, and post-injury response to rehabilitation [5].

The Achilles tendon (AT) is one of the most commonly injured tendons [2, 3, 6]. Anatomically, the AT is composed of the tendons of the lateral gastrocnemius, medial gastrocnemius, and soleus muscles, and is among the thickest and strongest tendons in the body; these features enable the AT to resist large tensile forces. However, the anatomical location and AT ruptures have increased over the past four decades and continue to rise [7]. As the AT connects the soleus and gastrocnemius muscles to the calcaneus bone and is vital to mobility, injury to the AT can result in severe debilitation in affected patients [7]. Injuries can lead to pain, swelling, loss of function, and immobility, which may result in decreased daily activity and restrictions on sports activities [8]. The severity of Achilles tendinopathy can be measured based on the severity of pain, alterations in daily living activities, and limitations or elimination of sporting activities [9]. Furthermore, severe AT injuries can be classified for athletes according to the resulting loss of function, > 30-day time loss, and presence of surgical injury [10].

Current treatments for tendon injuries include conservative methods such as pain management, physiotherapy, cryotherapy, and rehabilitation exercise strategies. If these strategies fail or are not indicated because of the cause and/or severity of the tendon injury, there are surgical treatment options that include direct suturing, implantation of tendon grafts (autografts, allografts, xenografts), implantation of synthetic materials, and the use of biologic therapies (cellular, acellular) [5]. However, current methods and therapies often result in clinical failure, leading to reinjuries, scar tissue formation, and difficulties in restoring functional and biomechanical properties [11]. For example, in a study involving 762 patients, up to 3.7% of surgically managed AT injuries failed to fully recover, and up to 9.8% of conservatively managed ATs failed to return to normal function [12]. Therefore, developing a functional, practical, and lasting treatment for tendons is necessary.

Tissue engineering is emerging as a promising field for AT research. It involves stem cells, biologics, and scaffolds made from natural, synthetic, or composite materials. The goal is to develop a construct that restores tendon functionality by mimicking both the mechanical and biological characteristics of the tendon. This approach aims to recreate the AT microenvironment and enhance healing by utilizing stem cells and biologics. This review investigates tissue engineering constructs for AT injuries, including their components—cells, biologics, and material scaffolds—as well as their biomimetic features and translation to the clinic.

### Achilles tendon anatomy, composition, and biomechanics

The AT is a connective tissue that is located in the posterior aspect of the lower leg and connects the gastrocnemius and soleus muscles to the point of the calcaneus bone. The tendon components that comprise the AT originate from the posterior aspect of the leg, arising from the lateral gastrocnemius, medial gastrocnemius, and soleus muscles, which insert onto the point of the calcaneus bone. The anatomical locations of AT injuries may be described based on their location: intramuscular region, non-insertional region (middle tendon region), and pre-insertion site (about 2 cm above the calcaneus) [13].

Each tendon in the AT is composed of highly organized fiber bundles encased in a thin layer of connective tissue called the epitenon. These bundles include primary, secondary, and tertiary fibers, all composed of collagen fibers formed from fibrils with chains of tropocollagens. [14] The fibrils form tendon fascicles, which are bundled together by the endotenon layer, and this entire structure is covered by the epitenon layer [15]. These layers allow the vasculature to traverse and supply nutrients. In addition, unlike most flexor tendons of the hand and foot, the AT is not housed within a synovial sheath at any point along its course [16]. The AT primarily consists of Type I collagen (60–85%); it includes lesser quantities of collagen types III, IV, V, and VI; and contains elastin fibers and proteoglycan content [17]. Tenoblasts and tenocytes, constituting 90–95% of cells within the tendon, secrete the extracellular matrix (ECM), which contains non-collagenous glycoproteins such as fibronectin, laminin, thrombospondin, and tenascin-C [18].

The AT is the strongest, largest, and thickest tendon in the human body, having a length of about 150 mm, a thickness of 5 to 7 mm, and a width of approximately 20 mm [19]. Biomechanical assessment of the AT demonstrates tissue properties, including tensile strength, Young’s modulus, and elasticity. The AT transmits the strongest forces across the ankle to extend the foot [20]. The characteristics of the biomechanical forces exerted in the typical AT in people includes: average ultimate tensile stress of 100 MPa; the Young’s modulus range 1 to 2 GPa; range for strain at failure is 4–10%; stiffness between 17 to 760 N/mm; and elasticity around 6% of input energy during the load-bearing phase of walking [13, 21]. These measurement results can vary based on the analysis methods used, such as ultrasound force and location [13], as well as individual factors like age, gender, height, weight, physical condition, and activity level.

### Achilles tendon defect, healing and repair: mechanism and current methods

Healing remains a major clinical challenge for AT injuries, mainly due to the hypovascular and hypocellular nature of the tendon, which, as noted above, limits its intrinsic capacity for healing and repair. Indeed, during the healing process, AT injuries are often complicated by maladaptive outcomes collectively referred to as tendinopathies. These include the formation of non-tendinous tissues—such as mineralized deposits, cartilage-like tissue (chondrogenesis), and fatty infiltrates (adipogenesis)–as well as the development of fibrotic tissue, adhesions, and scarring. Collectively, these pathological remodeling events disrupt the normal composition and architecture of the extracellular matrix (ECM), compromising the tissue’s ability to restore its native mechanical and functional properties. The outcome often involves incomplete or poor healing, characterized by diminished tensile strength, altered elasticity, and an increased risk of reinjury or chronic pain.

Successful healing of the AT requires more than just structural closure of the injury site; it demands the re-establishment of tendon-specific cell populations (e.g., tenocytes and tendon stem/progenitor cells), the restoration of aligned collagen fibril architecture, and the recovery of biomechanical performance to pre-injury levels. Achieving this balance between biological repair and mechanical resilience is essential to ensure long-term functional outcomes and to reduce the risk of recurrence. Due to these complexities, AT healing is increasingly recognized as not simply a reparative process but a dynamic interplay between cellular, molecular, and mechanical factors, underscoring the need for advanced therapeutic strategies that can guide tissue regeneration toward a true tendon phenotype.

The ECM of the AT, like any other tissue, is a complex network of biological molecules, including predominantly collagen I and other components like proteoglycans and glycoproteins. The ECM provides the tissue signature and support and transmits forces from the muscle to the tendon, contributing towards its normal function. It has been established that the force transmission of the muscle–tendon complex is dependent on the structural integrity between the muscle fibers and the ECM. It is important to note that the arrangement, orientation, density, and length of the collagen and muscle fibers are crucial to the function of the tendon. Hence, tendons are one of those tissues wherein the ECM is dynamic and adapts to the functional needs. This dynamic nature, to some extent, involves turnover in collagen proteins to meet the changes experienced in mechanical loading or inactivity and disuse of the tendon [17, 22].

AT healing occurs in three phases: inflammation, proliferation, and remodeling [23]. These healing phases involve various cells from multiple sources, including inflammatory cells, resident fibroblasts, and tendon or marrow-derived mesenchymal stem cells [24]. When the AT ruptures, an immune response is triggered involving the recruitment of macrophages, neutrophils, and monocytes to the injured tissue [23]. The inflammatory cells release pro-inflammatory cytokines, initiate the repair process, and release growth factors that promote the recruitment of cells, including tendon fibroblasts and mesenchymal stem cells [24]. Additionally, tenocytes adjacent to the site of injury undergo apoptosis, and surrounding progenitor cells and tenoblasts assist the healing process by migrating, proliferating, and differentiating [25]. Following injury, ECM production increases markedly and has a significant role in remodeling the AT. During the repair phase, collagen type III is the most dominant during remodeling [26]. Although specific cellular events and ECM composition may vary depending on the physiology of a given tendon injury, whether acute or chronic – with acute injuries typically characterized by a strong inflammatory response and rapid ECM deposition, and chronic injuries often showing extended inflammation, fibrotic ECM accumulation, and impaired remodeling – the main factors guiding regeneration remain the resident cells, secreted cytokines, growth factors, and the ECM [24, 27].

The natural healing of the AT is relatively slow and often does not fully recover to achieve complete functionality and strength compared to normal tendons [28]. To address this problem, various strategies have been applied: conservative treatment, surgical treatment, and regenerative approaches (cell and growth factor therapy). Conservative methods which are typically non-surgical approaches that reduce pain and inflammation, protect the tendon from further injury, and support the endogenous tendon repair process. These include cast immobilization and functional bracing with early rehabilitation [29]; cryotherapy [30]; extracorporeal shockwave therapy (ESWT) [31]; therapeutic ultrasound [32]; electrotherapy [33], and eccentric exercises [34]. These approaches are usually indicated for mild injuries, and operative methods may be used if conservative methods fail, e.g., after 3 to 6 months, or for more severe cases [35]. In contrast, surgical methods are used when non-surgical, conservative methods are ineffective or cannot be used. Broadly, the approaches include removal of the injured segment of the tendon, primary repair (suture reconstruction) of the AT, or implanting grafts such as autografts (tissue from the patient), allografts (tissue from a human cadaver), and xenografts (tissue from an animal carcass). However, grafts have limitations, such as increased risk of fibrosis, loss of tissue functionality, and failure to restore normal tendon properties. Additionally, autografts have limitations due to donor site morbidity [36], as well as increased likelihood of pain at the harvest site, loss of function in donor site tissues after harvest, and limited graft dimensions that may not meet the requirements of the injury. Limitations and concerns of alternative tendon grafts include graft failure, rejection, infection, and disease transmission [37]. As a result, the functional outcomes linked to tendon grafts are generally poor [38, 39]. Although there are disadvantages to the current surgical methods for AT treatments, operative treatments have fewer post-operative infections and fewer re-ruptures compared to non-operative treatments [40].

Growth factors and other biologics have emerged as valuable treatments for some Achilles injuries through cell-free approaches that involve injecting substances such as platelet-rich plasma (PRP) and platelet-rich fibrin (PRF), all derived from the patient’s blood and containing various beneficial growth factors [41]. PRP has become a commonly used treatment in clinical practice, including the treatment of AT injuries. Autologous PRF matrices showed promising results after surgical procedures with faster functional recovery in 6 athletes [42]. Similarly, a prospective randomized controlled clinical trial showed similar results after surgical procedures using the end-to-end Krackow suture method, indicating that functional performance was better in the PRP group [43]. While these studies suggest that PRP and PRF treatments improve postoperative functional recovery, their sample sizes are relatively small. To overcome this limitation, larger-scale trials are needed to validate these findings. In a randomized controlled trial using PRP without surgical AT treatment, multiple PRP injections did not show significant tendon healing or improve the outcomes (patient-reported, functional, or clinical) [44]. This study indicates that using PRP alone or alongside surgical treatment can impact the outcome. Growth factors such as TGF-β [45], BMP [46], IGF [47], FGF [48], PDGF [49], and VEGF [50] have been individually studied and applied in tendon regeneration. They play crucial roles in repair and regeneration by recruiting and differentiating cells, synthesizing ECM, and promoting angiogenesis [51]. Among their essential roles and advantages, they have short, effective half-lives and are volatile [51].

Current research in this area is increasingly exploring the potential of cell-based therapies for treating AT injuries, using cells sourced either from the patient’s own body (autologous) or from other donors, including allogenic or xenogeneic sources. The cell types used in these therapies can be stem cells, such as mesenchymal stem cells, or differentiated cells, such as tenocytes [52]. Tenocytes are abundant cells in the tendon microenvironment, playing a crucial role in tendon healing. They create the microenvironment by secreting specific growth factors and rebuilding the ECM during healing. Furthermore, stem cells are extensively studied for their potential to repair and regenerate various tissues. This is due to their multipotent or pluripotent features, which allow them to differentiate into various cell types. Different types of stem cells have been studied for AT regeneration, including tendon-derived (TDSCs) [53], adipose-derived (ADSCs) [54], bone marrow-derived (BMSCs) [55, 56], and induced pluripotent stem cells (iPSCs) [57]. Some stem cells, including mesenchymal stem cells, possess properties beyond their multipotency, including immunomodulatory and self-renewal capabilities. Although MSCs offer benefits, stem cells must be properly characterized and tested before clinical use. Particularly, the allogenic and xenogeneic stem cells, which are not derived from the patient’s tissues. Transplanting these cells may result in adverse effects, such as rejection through donor-host disease transfer or graft failure. Concerns regarding the use of pluripotent stem cells can include the risk of forming teratomas, necessitating further research to identify an optimal method for ensuring their viability and safe use in cell therapies [58].

### Tissue engineering of the Achilles tendon

Tissue engineering is an emerging field that aims to improve the regeneration of the AT using various combinations of cell therapy, biological therapy, and biomaterial scaffolds for guided tissue regeneration. During AT healing and regeneration, the quality of tissue repair is influenced by biomechanical forces, tissue structure, endogenous cells, and ECM composition [59]. The goal of Achilles tissue engineering is to optimize tendon healing and repair. This involves using biomaterial scaffolds to provide functionality, as well as incorporating cells and growth factors to create a microenvironment that supports tendon healing.

Tissue engineering is a multidisciplinary field combining expertise from material science, biomedical engineering, and cell biology to develop constructs for tissue repair and regeneration [2]. Understanding tissue characteristics and biomechanics is essential for designing a scaffold that accurately reflects tissue properties, mimics tissue architecture, and restores functionality [60]. One approach to tissue engineering for guided tissue regeneration involves the creation of scaffolds that facilitate the regeneration. These scaffolds can also serve as delivery vehicles for cells, growth factors, or other bioactive particles. To achieve this, various manufacturing methods can be employed, including 3D printing, electrospinning, electrospray, surface deposition, and molding [61, 62]. The selection of materials, solvents, and scaffold fabrication methods influences their properties throughout the entire process. Optimizing polymers, material preparation methods, and manufacturing techniques is essential for advancing guided tendon regeneration. Once these are determined, scaffolds can be developed and tested in vitro to characterize their mechanical properties before they are introduced into animal testing for feasibility and validation studies. This process helps characterize the material and provides an opportunity for further modifications. Following material development, cells and biologics can be introduced to the scaffold in vitro to create an enhanced construct for tissue engineering. Cells and biologics have a crucial role in establishing a tissue-specific microenvironment that serves to create an idealized healing environment for tissue repair and regeneration. The selection of materials, cell types, and beneficial biologics is based on the specific 3D matrix with ECM required for tissue regeneration. Following in vitro cytocompatibility testing, in vivo biocompatibility evaluations are essential to assess whether the scaffold causes cell stress or apoptosis, potentially leading to adverse reactions in the body. Additionally, functionality tests must confirm that the implanted construct performs effectively in vivo.

#### Biomimetic constructs

Tendon tissue engineering has used different polymers to develop scaffolds. These polymers are classified into three categories: natural, synthetic, and acellular. Natural polymers are derived from natural sources and utilized in tissue engineering constructs. For example, alginate is a natural polymer derived from the cell walls of brown algae and is used in various regenerative constructs. Additionally, synthetic polymers are created artificially and are commonly used in tissue engineering because they can be tuned and modified to achieve specific desired properties. Furthermore, acellular matrices containing ECM proteins are derived from tissues that are decellularized and processed using special techniques. These matrices can be used as scaffolds to mimic the target microenvironment and are designed to mimic the AT structure. These various polymers can be used alone or combined to create a composite biomimetic scaffold that mimics the properties of AT. Composite scaffolds are then combined with biologics or different cell types to develop biomimetic constructs that enhance tissue repair and regeneration.

#### Natural materials

Natural polymers are commonly used in tendon tissue engineering due to their biocompatibility and bioactivity. Materials such as collagen, hyaluronic acid, fibrin, chitosan, and cellulose are natural polymers and can be applied individually or as composites [63], though achieving the required mechanical strength, particularly for the AT, remains challenging. Nonetheless, numerous studies demonstrate that scaffold composition and architecture can significantly improve both biological and mechanical outcomes. For example, silk fiber scaffolds, filled with silk fibroin/collagen [64] or aligned collagen fibers, produced by advanced extrusion techniques [65], closely mimic the native tendon’s microstructure and show enhanced in vivo cell response, differentiation, and functional recovery. Due to enhanced biomimicry from aligned fibers, the scaffolds combined with BMSCs exhibited higher tensile mechanical properties, including maximum tensile stress and Young’s modulus, compared to random fibers, 5.02 ± 0.18 vs 1.92 ± 0.17 MPa and 34.52 ± 8.19 vs 10.51 ± 1.42 MPa. These findings demonstrate that not only are the materials used in constructs and their tunability important, but also the way the materials are manufactured, and their architecture must be considered, as this significantly influences mechanical properties, cell response, and functionality. Another study compared chitosan-based asymmetric scaffolds seeded with tendon stem/progenitor cells (TSPCs) with the empty defect and showed reduced peritendinous adhesion, while promoting cell proliferation and tenogenic gene expression, leading to improved mechanical strength (significantly higher Young’s modulus compared to the empty defect) and collagen fiber alignment in animal models [66]. These results demonstrated that the chitosan scaffold, particularly when seeded with TSPCs, enhanced tendon regeneration, showing promise as a therapeutic strategy for AT repair. Another silk fibroin scaffold, created using a combined micellization and fibrillation process to mimic tendon structure with highly oriented nanofibrils, achieved strength (6 ± 1 MPa) and stiffness (12.0 ± 1.3 MPa) that closely match those of the AT [67].

Novel approaches are being explored for Achilles tissue engineering, including ostrich eggshell membrane (ESM) combined with PRP. This combination promoted collagen synthesis, collagen organization in newly formed tissues, and gait recovery in vivo [68] (Table 1). However, the group with only the ESM scaffold, without PRP, showed a higher inflammatory response, greater calcification, and the lowest mechanical properties. Additionally, ESM combined with PRP demonstrated increased ultimate (a.k.a. breaking or failure) strength (48.91 ± 15.56 MPa), but decreased Young’s modulus (20.90 ± 7.53 MPa) compared to the normal tendon, while the measurements between ESM and ESM + PRP did not show significant differences. Furthermore, a novel bioactive hydrogel was produced from salamander skin secretions, and it exhibited both antibacterial and antioxidant properties; the hydrogel reduced peritendon adhesion but did not eliminate it [69]. Moreover, the regenerated tendons in rats showed a modulus closer to normal tissue, but their ultimate tensile strength remained weaker (7.1 ± 1.9 MPa) than that of the normal tendons (14.0 ± 5.8 MPa). There has been significant progress in developing hydrogels that trigger gelation at body temperature when injected, known as thermosensitive hydrogels. For example, in a study, researchers demonstrated that a thermosensitive chitosan/collagen hydrogel combined with MSCs supported regeneration in the AT injury model by promoting anti-inflammatory and pro-repair signaling pathways [70]. In addition to their improvements, the hydrogel + MSCs group showed enhanced tensile strength in a rat model, close to that of the uninjured tendon, and higher than that of the hydrogel-only group.

**Table 1:**
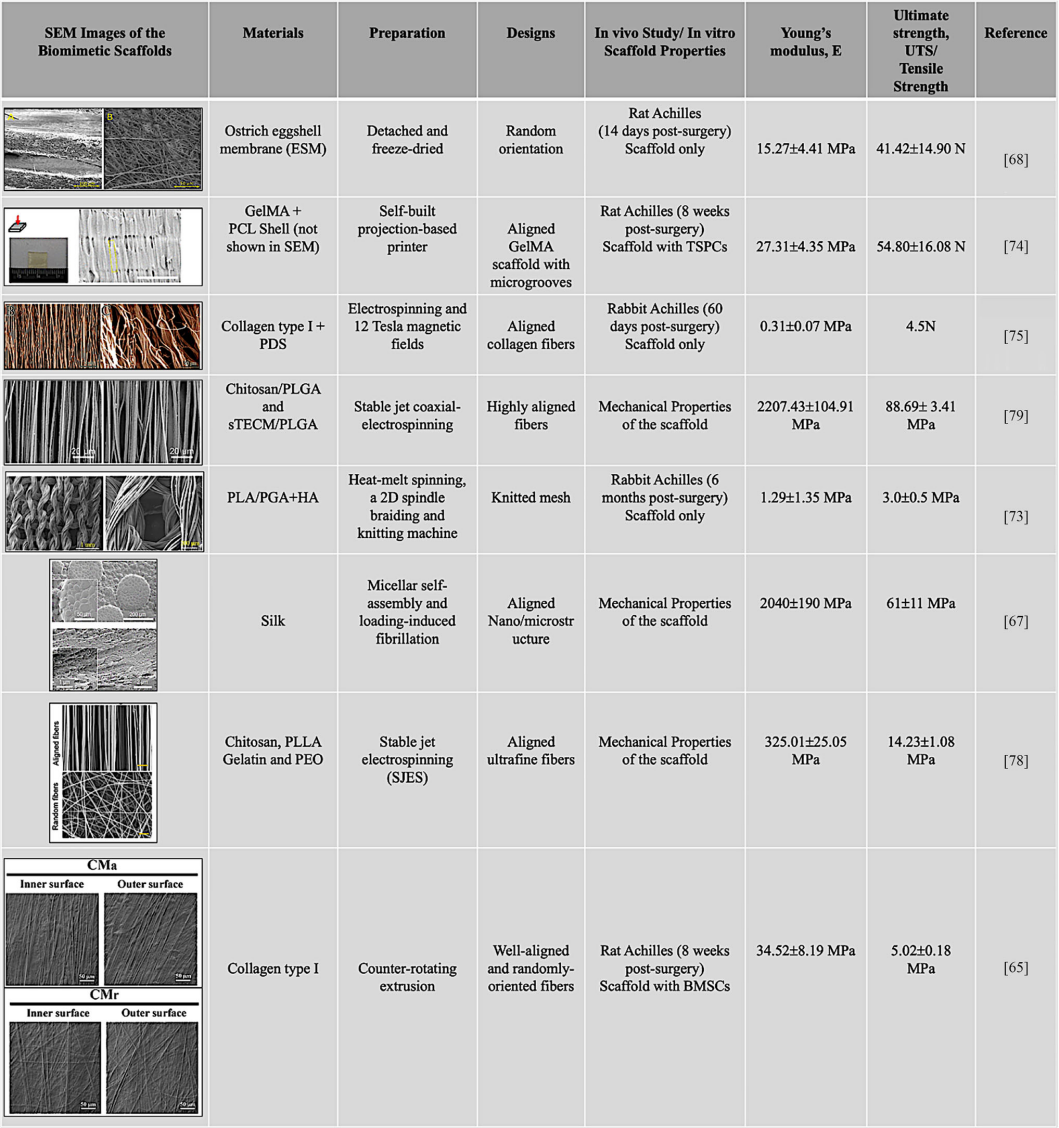
Different materials, including natural, synthetic, or composite, along with various designs and fabrication methods, like printing techniques and the mechanical properties (Young’s modulus, E and Ultimate/Tensile strength, UTS) are employed to replicate the aligned structure and create biomimetic constructs for Achilles tendon regeneration (Permissions have been obtained for the use of images from published articles)

Taken together, these advances underscore that the success of tendon regeneration depends not only on the choice of natural polymer but also on scaffold design, fiber alignment, and incorporation of biological cues. While many constructs demonstrate improved mechanical properties and cellular responses in preclinical models, further work is needed to refine these biomaterials for consistent translation to clinical practice.

#### Synthetic materials

Synthetic polymers are widely used in scaffold design for regenerative medicine due to their tunable properties and ability to mimic tissue biomechanics. Materials such as poly(lactic-co-glycolic acid) (PLGA), polylactic acid (PLA), polycaprolactone (PCL), and polyglycolide (PGA) are tested for tendon engineering with attention to biodegradability, toxicity, cytocompatibility, biocompatibility, and biomechanics. Scaffold development follows a staged process from preparation to in vitro and in vivo testing.

Recent advances highlight the value of piezoelectric polymers for their anti-inflammatory properties and aligned scaffold structures for mimicking AT that influence cell response. For example, piezoelectric PCL combined with tetragonal barium titanate nanoparticles (BTO) promoted anti-inflammatory effects, collagen deposition, and functional recovery in AT models [71]. In contrast, disorganized collagen structures, immature collagen, and fibrotic scar tissue were observed in PCL groups. Also, mechanical properties such as tensile strength, Young’s modulus, and maximum load were higher in PLC/BTO (24.66 ± 1.02, 49.59 ± 2.90 MPa, respectively) than in PCL alone (17.81 ± 0.85, 40.93 ± 3.75 MPa, respectively). These findings demonstrate the potential of piezoelectric nanofilms containing BTO to promote tendon regeneration. Another piezoelectric polymer, polyester-based elastomers, enhanced biomechanical strength with an elastic modulus as low as 0.3 MPa and recoverable strain up to 300%, enabling it to withstand Achilles’ motion [72]. They suggested that the scaffold reduced the risk of re-rupture by improving biomechanical properties and demonstrating biocompatibility, with no inflammatory heat response around the implantation site. While another study with hybrid PLA/PGA scaffolds modified with hyaluronic acid also showed improved mechanical performance, it also demonstrated reduced inflammation, highlighting the promise of cell-free regeneration strategies [73]. However, they also demonstrated immaturity of regenerated tendons and limited improvement in mechanical properties such as Young’s modulus (1.29 ± 1.35) and strain at break.

Despite cell-free scaffolds, stem cells can be triggered to differentiate into native cells for healing, facilitated by the scaffolds’ aligned or biomimetic topographies (Table 1). Since tendons have a highly organized collagen fiber structure, researchers attempt to mimic the micro- and macro-tendon structure. For instance, a composite synthetic scaffold with parallel grooves was printed using GelMA (core) and PCL (shell), enhancing mechanical properties, cell alignment, tenogenic differentiation, and reducing inflammation compared to a flat surface [74]. In addition, the scaffold + TSPCs group showed better mechanical properties (failure force: 54.80 ± 16.08 N, modulus: 27.31 ± 4.35 MPa) and regeneration than the scaffold-only group, and a higher Achilles Functional Index (AFI) score than the scaffold-only group. A study also demonstrated that a biomimetic composite—comprising the scaffold wrapped with polydioxanone (PDS), similar to peritenon encasing a tendon—shows enhanced biomechanical properties and promotes tendon regeneration, as PDS is biocompatible and an appropriate choice for mimicking peritenon due to its slow absorption rate [75]. Another bioinspired scaffold design used poly-L-lactic acid (PLLA) and polyethylene oxide (PEO) to develop micro-nanofiber electrospun scaffolds to mimic the fibrils and fibers in AT [76], and micro-nanofibers have shown improved collagen organization, tendon marker expression, and tensile properties, such as the stress at failure (6.12 ± 1.35) compared to the control group, leading to superior regeneration outcomes.

Overall, synthetic scaffolds are advancing both mechanical reinforcement and biological functionality. Future work must focus more on long-term biodegradation and biocompatibility to enable translation of these findings into clinical applications.

#### Composite materials

Polymer composites are increasingly advancing tissue engineering by combining natural and synthetic polymers, acellular matrices, or both, to achieve a balance between biological function and mechanical properties. Recent research highlights a variety of strategies for AT regeneration.

One notable study developed a chitosan piezoelectric gel (CSPG) that combines natural chitosan with a synthetic piezoelectric elastomer to provide both biological and mechanical support by improving load to failure and elastic modulus, mimicking the AT structure [77]. Additionally, low-intensity pulsed ultrasound (LIPUS), a conventional technique in orthopedics, was also used to accelerate healing. The combination of CSPG and LIPUS was found to enhance cell viability, proliferation, migration, antibacterial activity, and tenogenic differentiation. These effects are attributed to electric fields generated under ultrasound stress. This research demonstrates that combining different polymer types in a composite construct, along with integrating treatment methods in orthopedics, can enhance healing outcomes. However, the CSPG group still shows some disorganized morphology, with more cells and blood vessels than the sham group. Another approach involved creating a composite scaffold from chitosan, PLLA, gelatin, and PEO via stable jet electrospinning, producing ultrafine, aligned fibers that mimic the tendon microstructure [78] (Table 1). Human iPSCs differentiated into MSCs and then into tenocytes on these fibers, demonstrating superior mechanical strength in vitro compared to randomly oriented fibers, with higher tensile strength (14.23 ± 1.08 vs. 2.43 ± 0.28 MPa) and Young’s modulus (325.01 ± 25.05 vs. 74.46 ± 12.99 MPa). The construct also demonstrated biomechanical measures such as stiffness, stress at failure, and Young’s modulus, as well as tendon-specific gene expression in vivo, most of which indicate improved Achilles recovery; however, it also showed slightly lower strain at the breaking point with aligned fibers compared to random.

Further advancements include Tu et al.’s fabrication of highly aligned chitosan (CTS)/PLGA and tendon-derived extracellular matrix (TECM)-CTS/PLGA fibers using coaxial electrospinning to create scaffolds [79] (Table 1). The TECM-modified fibers enhanced cytocompatibility and tendon marker expression, leading to in vivo regeneration with collagen structure comparable to that of native tendons. However, they also mentioned the limitations of the study, such as TECM attenuating the mechanical properties of the scaffold (Young’s modulus without TECM: 88.69 ± 3.41 vs with TECM: 64.11 ± 2.81 MPa), which may affect further studies. They have observed abnormal calcification after 12 weeks post-implantation, and the need for verification using human cells and large-animal testing for clinical application. Similarly, TECM loaded with tendon-derived stem cells and embedded in a dual-phase PCL/silk fibroin scaffold showed enhanced cytocompatibility and ECM restoration [80]. The scaffold combined with TDSCs promoted well-aligned collagen fibers and a higher tensile modulus (55.95 ± 22.04 vs 108.5 ± 6.48 MPa) compared to the scaffold without TDSCs (41.92 ± 16.36 vs 73.60 ± 9.34 MPa), at 6 and 12 weeks, respectively, leading to better tendon development and maturation in vivo. They also noted that the construct did not fully recover its mechanical properties within 12 weeks and exhibited poor fiber organization. Another natural-based approach combined silk fibroin, PLGA, and collagen to produce a composite scaffold with a weft-knitted mesh structure, unlike the aligned structure observed in previous studies [81]. This scaffold was seeded with MSCs, showing improved results compared to the control (without MSCs), with maximum load (138.47 ± 27.68 vs 88.46 ± 20.07 N), stress (18.75 ± 3.90 vs 11.80 ± 2.71 MPa), and elastic modulus (130.49 ± 34.45 vs 82.56 ± 21.33 MPa) in rabbit models after 8 weeks, and with aligned collagen fibers resembling those of intact AT. This shows lower mechanical properties of the scaffold in the absence of MSCs. They also observed a small amount of inflammatory cell infiltration after 8 weeks, which disappeared at 16 weeks, and the material was gradually degraded. Additionally, they noted that tissue-engineered tissue formation was observed only at 16 weeks, not before, and that biomechanical properties should be tested beyond 16 weeks, highlighting the importance of long-term animal studies for clinical translation. Collagen remains a widely used natural polymer, and was also used in a collagen sponge within Vicryl mesh seeded with bone marrow MSCs, which restored stiffness to 98% and achieved a Young’s modulus (~ 70 MPa) significantly higher compared to the scaffold without MSCs (control) in vivo [82]. Furthermore, histological organization and elongated tenocytes were observed after three months, and the scaffold was entirely replaced with mature tendon tissue resembling intact tendons after six months. In contrast, the controls showed fibrotic repair with disorganized collagen fibers and limited regeneration. This study, like most others, highlights the importance of cells in regenerative constructs for improved Achilles repair.

Overall, natural components promote biocompatibility and favorable cell responses, while synthetic materials enhance mechanical properties. The continued development of composite scaffolds holds great promise for AT regeneration. Further research should focus on improving surgeon-friendly handling and conducting long-term validation of biological and mechanical performance to support clinical translation.

#### Acellular materials

Acellular materials are scaffolds derived from native tissues through decellularization, a process that removes cells while preserving the ECM [83]. For AT repair, these materials are particularly valuable because they retain the biochemical features, structural organization, and mechanical framework of tendon tissue, creating a microenvironment that supports regeneration and function. In short, by mimicking the native ECM, acellular scaffolds promote cell attachment, proliferation, and differentiation, while reducing immunogenicity compared to tendon grafts. When combined with stem cells or growth factors, they further enhance tendon-specific healing, offering a promising strategy for functional and biomechanically robust AT regeneration. Tissues are typically chosen to match the target application – for AT repair, tendon tissue is decellularized to retain its native ECM composition. For example, rat tail tendons processed by freeze–thaw and combined with adipose-derived stem cells (ADSCs) showed good biocompatibility, low immunogenicity, and upregulation of tendon-specific markers [22]. In vivo studies revealed improved gait recovery and collagen deposition, and biomechanical tests found a significant benefit of ADSCs combined with the scaffold compared to scaffold or ADSCs alone. This study also suggested that local inflammatory microenvironments and growth factors regulate the differentiation of cells implanted at the site, and that immune rejection of the transplants warrants further investigation. As shown, the recellularization of decellularized scaffolds can enhance the biomechanical properties of constructs. Another example: Bottagisio et al. compared autografts (the gold standard), decellularized equine xenografts (DG), and recellularized tissue-engineered xenografts (TEG) with autologous bone marrow-derived mesenchymal stem cells (BMSCs) [84]. Implanted in rabbit AT, TEGs produced outcomes similar to autografts, with enhanced biomechanics (elastic modulus and failure stress), increased collagen deposition, reduced inflammation, and improved biocompatibility, compared with DGs, highlighting the immunomodulatory role of BMSCs. TEGs also demonstrated active remodeling through the expression of proteoglycans and matrix metalloproteinase (MMP) activity, suggesting their promise as an autograft alternative.

Novel sources are also being explored, including decellularized tilapia skin (DTFS), which offers low biological risk and ethical advantages over mammalian ECM; and crosslinking (C-DTFS) with improved stiffness and Young’s modulus, while tensile stress testing showed different results with different skins and according to their wet/dry conditions [85]. C-DTFS also supported cell viability and tenogenic differentiation of TDSCs. In rat models, C-DTFS + TDSCs achieved functional outcomes with reduced scarring and collagen organization comparable to autografts, outperforming acellular scaffolds, demonstrating the application of these improved engineered-based approaches. Although they did not mention immunogenic components, they noted that DTFS had limited antibacterial activity and may be coated to prevent postoperative infections, suggesting it for further study. In addition to their limitations, they observed some cartilaginous tissue at 8 weeks post-operation in the C-DTFS and defect groups.

Overall, work in this area has provided further evidence and clinical support that acellular scaffolds mimic the tendon microenvironment and support positive host responses [86, 87]. Even though some of them test their constructs’ immunogenicity and discuss, most do not show any immunogenic reactions, suggesting that further discussion of their immunogenicity can be helpful for the literature and eventually lead to clinical translation. Additionally, the recellularization with stem cells consistently demonstrated enhanced biomechanical and regenerative outcomes [88]. However, further studies are needed to confirm long-term biomechanical reliability and ensure consistent clinical applicability of decellularized tissue.

**Fig. 1:**
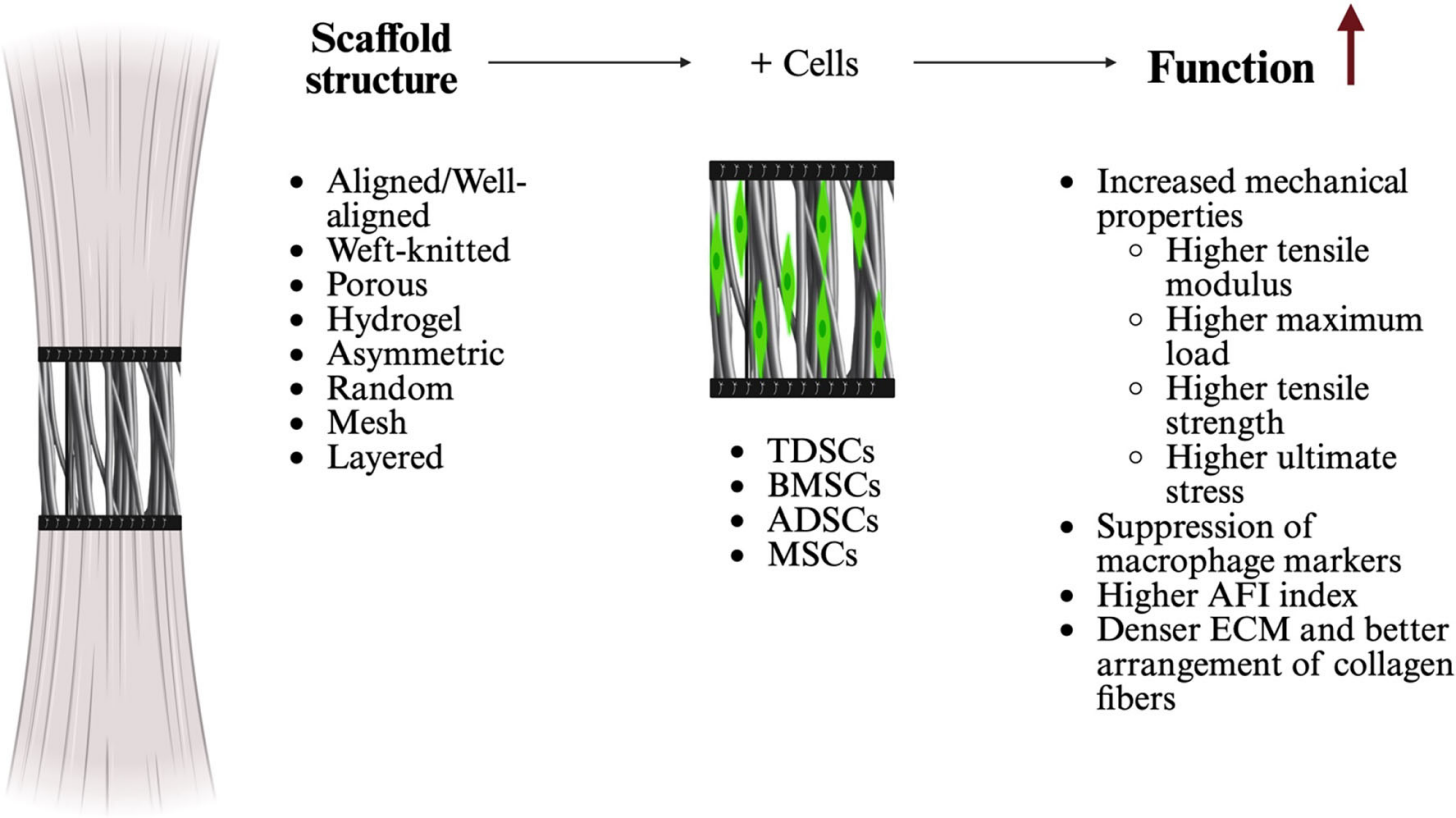
Schematic to illustrate the structure – function relationship between scaffolds, cells and functional outcomes important for tendon tissue engineering. The functionality of the regenerative constructs depends on several factors, such as scaffold structure and cells within the constructs. Combinations of these factors can create functional constructs, with a goal of enhancing at least one of the listed features. The listed structures, cells, and functionalities were taken from the literature cited in this review article. (Image was created using Biorender)

Considering all material types, scaffold designs, and cellular and biological cues, the main goal is to develop a functional construct that matches the Achilles tendon’s different aspects, including its mechanical properties, structure, cellular composition, and protein expression. Achieving optimal results across all these factors is challenging. As reviewed in this article, the effectiveness of Achilles regenerative constructs depends on many elements, but those incorporating cells generally yield better outcomes. The scaffold structures and cell types illustrated in Fig. 1 have been combined in various ways in the literature, with many studies reporting functional improvements compared to controls, which had either no scaffold or only a scaffold. This review emphasizes that it is not solely the scaffold’s structure that influences functionality, but rather its combined interaction with cells, which respond to the topographies, geometries, and architectures of the scaffolds.

### Clinical translation

**Fig. 2:**
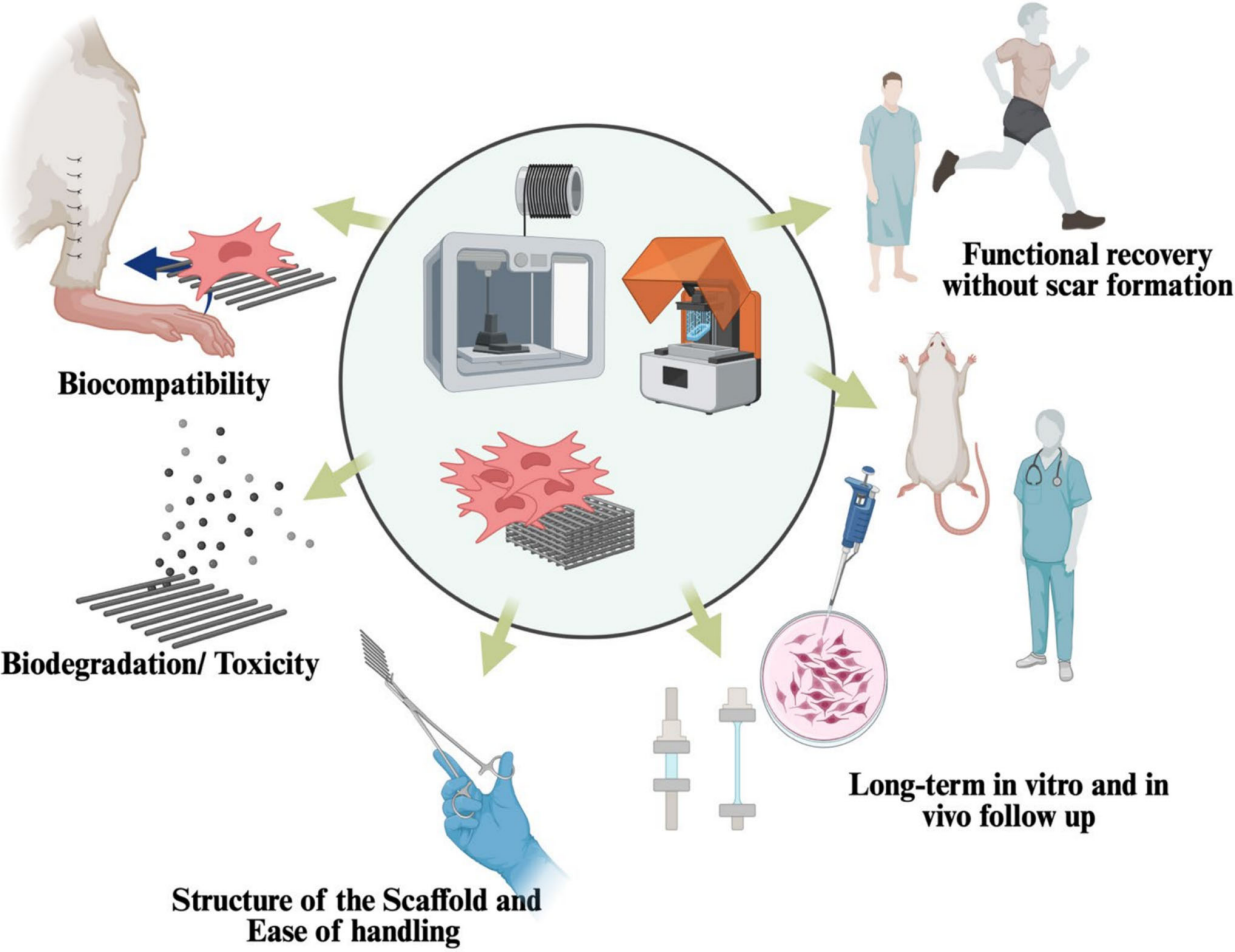
Key considerations for translating Achilles tendon constructs from bench to clinic. These include establishing clear and consistent outcomes ensuring functional recovery. (Image was created using Biorender)

Most studies on Achilles regeneration test the constructs in vitro and in vivo. They are also investigating cell-based vs. cell-free approaches to determine which can provide greater clinical advantages. For clinical applicability, the constructs tested across multiple parameters are essential, such as increased mechanical properties and functionalities of the scaffolds when combined with cells (Fig. 1). Although numerous published and upcoming studies aim to enhance Achilles tendon regeneration and demonstrate functional outcomes, their translation into clinical practice remains challenging. In addition to their in vitro or in vivo functionality, several criteria must be met for the constructs to be applicable for treating Achilles defects (Fig. 2). For instance, structures, biocompatibility, biodegradability, toxicity, scaffold handling by surgeons, and long-term in vitro and in vivo follow-up. One of the least discussed criteria is the surgeon’s handling of the scaffolds. Even though it may seem simple and unimportant, this parameter plays a significant role in the clinic, enabling scaffolds to be prepared, stored, and used easily. For example, the scaffolds need to be designed with consideration of placement in the defect area, such as with suturing. Suture sites can be integrated into the scaffold to stabilize it, as demonstrated by Jiang. et al. for rotator cuff tendon [89]. Figure 1 also demonstrates that suture sites can be placed on both sides of the scaffolds for Achilles tendon surgeries. Furthermore, medical doctors, biologists, chemists, engineers, and regulators are required to oversee and facilitate all these parameters mentioned in Fig. 2, making the constructs applicable in clinical settings. To make these multidisciplinary developments translational in human medicine, the constructs are tested in animals or using bioreactors and organ-on-a-chip technologies. This section will mention in vivo applications of constructs for AT, their clinical translation, and current human practices.

#### In vivo models for clinical translation

While in vitro studies clarify the cytotoxic properties of the final tissue-engineered product, in vivo investigations will help not only in healing damaged tissue but also in understanding the biocompatibility, biomechanics, and function of the tissue-engineered construct. All in vivo studies performed thus far have been in animal models (rodents, rabbits, goats, and sheep) only. There is no known clinical trial ongoing to evaluate any 3D printed tendon construct. Thus, in vivo investigations continue to be of prime importance [90–92].

Rodents, primarily mice and rats, are commonly used as models in the development of new products. These models are cost-effective, and studies can be completed in a short time. However, the biggest drawback is that a clinically relevant situation that is observed in humans cannot be replicated in rodent models, and thus, repair techniques have been slow, or impossible, to implement. To circumvent these challenges, rabbits have emerged as an intermediate model, where the surgical techniques and functional outcomes can be evaluated to some extent. Large animal models, including horses and dogs, provide a much closer match to humans. These are required to translate a novel therapy from the bench to the clinic.

Simultaneously with the choice of the animal model, the choice of tendon injury or damage is made. Tendon injury can be induced by injections of specific reagents, such as collagenase, TGF beta, or prostaglandins, all of which are intended to stimulate inflammation and degeneration. These injection–based models are commonly used, and the concentrations of the inducers, the timing, and the biological pathways that are studied have been clearly documented. These models are primarily rodent-based, making it relatively easy to delineate the biological mechanisms of tendon damage and recovery. Longitudinal and transverse incisions are also used to induce tendinopathy. Similarly, other animal models, including trauma–injury–based models, overuse and mechanical loading models, are also described in the literature. Animal models not only serve to investigate the biological mechanism(s) of tendon injury and repair but also, help examine the tissue healing effects of novel biomaterials and 3D printed constructs in vivo.

Although various animal models and methods exist to establish Achilles tendon injury and create diseased models, the gap between animal models and clinical translation remains. There are several causes of this, such as differences between animal models and human Achilles (strength, mechanical function, and structure), a lack of a complete evaluation and understanding of cell function, scaffold properties, overall functionality, and material migration or degradation in vivo. For example, due to the limited number of animal groups and the short-term nature of animal studies, the studies cannot provide enough data to support long-term functionality from different aspects of the constructs. Also, not all studies can be conducted with larger animals for longer periods, such as Micro CT and histology. For example, these two require different sample preparation; thus, they need separate groups in the study. This increases the number of animals, the cost (especially for large animal models and rabbits), and decreases the study period (due to post-operative care). Given these challenges in animal studies, enhancing in vitro outcomes using dynamic systems (bioreactors or organ-on-a-chip) to test more parameters, eliminate groups, and extend study periods can be useful both before animal studies and for clinical translation.

#### Clinical approaches to human practices

Clinical approaches to human AT injury include medical (also known as conservative management), which involves the use of oral analgesics (e.g., acetaminophen, nonsteroidal anti-inflammatory drugs), rest, and immobilization (e.g., casting, bracing) of the affected joint [93]. Surgical approaches when conservative management is not possible include minimally invasive versus open procedures. A meta-analysis of the literature demonstrates reduced risk of re-injury in Achilles tendon injuries treated surgically [94–96]. Surgical approaches for an acute AT rupture include an open repair, minimally invasive incision repair, traditional percutaneous Achilles repair system (PARS), and the modified PARS using a suture anchor for augmentation [97]. Surgical repair of a chronic or neglected Achilles tendon rupture can include autografting, allograft, or xenografts using materials previously described. The goal of treatment is the same for both acute Achilles ruptures and chronic or neglected AT ruptures: restoration of anatomic length and physiologic tension, restoration of adequate strength, return to optimal function, pain reduction, and a reduction in complications. The goal, therefore, for engineered tissue materials should be to be functional, practical, and lasting, aligning with the surgical goals of Achilles rupture repair.

Interestingly, there are a variety of commercially available acellular dermal matrices, which are composed of the dermal layer and the extracellular matrix of human or animal skin in which the epidermal layer is removed. Matrix contains growth factors, collagen, and elastin. Even though these matrices are commonly used as skin grafts, they were originally designed to augment AT repairs. This is solely due to their mechanical strength and elastic properties [98]. For instance, a human derma matrix developed by Stryker, referred to as the Graft jacket, is proposed to reinforce tendon repair and allow for the growth of new tissue. The new tissue will grow through the graft material, which will allow for the tendon to grow stronger and more durable. The use as a skin graft is an atypical application. In either case, the matrices are expensive, they have a limited shelf life, there is no functional recovery, and most importantly, there is a patient–to–patient variation in the clinical outcomes. The majority of these commercial products are not investigated for their mechanism, and hence, as described in specific sections of this review article, it is imperative to understand the mechanism of action of these matrices prior to their use in the clinic. This strategy will significantly promote their clinical applications with improved outcomes.

### Future perspectives and conclusion

Tissue engineering is an evolving interdisciplinary field that combines biological and mechanical approaches, as well as the development and utilization of new scaffolds, tools, and methods, to better understand disease mechanisms and develop practical solutions. As discussed in the ‘In vivo Models for Clinical Translation’ section, there are limitations in regenerative medicine due to various aspects of the treatment methods and the complexity of the diseases. Because of these limitations, even though there are positive outcomes from each study, in vitro and in vivo, there is no clinical application of these constructs. To overcome these limitations, improving the tools currently used in this field and advancing the system’s ability to better understand the diseased environment are essential for bringing innovative solutions. Some of the latest approaches in regenerative medicine and tissue engineering utilize 3D cell culture systems, such as spheroids, organoids, and co-culture systems; advanced scaffolds using innovative techniques like laser micropatterning; and the development of dynamic systems such as bioreactors and organ-on-a-chip systems. For example, in organ-on-a-chip (OOC) systems, the goal is often to create a pathologic microenvironment that mimics a diseased environment to better understand the interactions within the microenvironment or disease pathophysiology, thereby finding effective solutions without the need for animal studies, or to improve the outcomes from the animal studies by decreasing the parameters and number of groups in the animal studies based on the OOC systems, and increasing the study period. As an example of the OOC system in tendon research, a study developed a human tendon-on-a-chip (hToC) and created a myofibroblast microenvironment (MME) to investigate the microenvironment of injured tendons, including the interactions between myofibroblasts and the vasculature and immune system, thereby demonstrating a platform for evaluating treatments for tendinopathies [99]. Another organ-on-a-chip study demonstrated guiding cell alignment on the tendon chip via magnetic manipulation to develop a tenogenic phenotype, utilizing both the intrinsic and extrinsic compartments of tendons in conjunction with perfusable vascular channels [100]. They created this setup to understand the pathologies of tendinopathy, such as tendon inflammation, and to test therapeutic strategies. These technologies and advanced cell and scaffold methods are emerging not only to provide insight into disease pathophysiology but also to develop more targeted and effective solutions and, ultimately, to create translational products.

## Data Availability

This is a review article and hence, does not have any research data to report. All published reports are appropriately cited.
